# NRT1.1B improves selenium concentrations in rice grains by facilitating selenomethinone translocation

**DOI:** 10.1111/pbi.13037

**Published:** 2019-01-09

**Authors:** Lianhe Zhang, Bin Hu, Kun Deng, Xiaokai Gao, Guoxin Sun, Zhengli Zhang, Peng Li, Wei Wang, Hua Li, Zhihua Zhang, Zihao Fu, Jinyong Yang, Shaopei Gao, Legong Li, Feiyan Yu, Youjun Li, Hongqing Ling, Chengcai Chu

**Affiliations:** ^1^ Luoyang Key Laboratory of Plant Nutrition and Environmental Ecology Agricultural College Henan University of Science and Technology Luoyang China; ^2^ State Key Laboratory of Plant Genomics Institute of Genetics and Developmental Biology Chinese Academy of Sciences Beijing China; ^3^ State Key Lab of Urban and Regional Ecology Research Center for Eco‐Environmental Sciences Chinese Academy of Sciences Beijing China; ^4^ College of Life Science Capital Normal University Beijing China; ^5^ State Key Laboratory of Plant Cell and Chromosome Engineering Institute of Genetics and Developmental Biology Chinese Academy of Sciences Beijing China

**Keywords:** NRT1.1B, selenomethionine, selenite, transport, rice (*Oryza sativa* L.)

## Abstract

Selenium (Se) is an essential trace element for humans and other animals, yet approximately one billion people worldwide suffer from Se deficiency. Rice is a staple food for over half of the world's population that is a major dietary source of Se. In paddy soils, rice roots mainly take up selenite. Se speciation analysis indicated that most of the selenite absorbed by rice is predominantly transformed into selenomethinone (SeMet) and retained in roots. However, the mechanism by which SeMet is transported in plants remains largely unknown. In this study, SeMet uptake was found to be an energy‐dependent symport process involving H^+^ transport, with neutral amino acids strongly inhibiting SeMet uptake. We further revealed that NRT1.1B, a member of rice peptide transporter (PTR) family which plays an important role in nitrate uptake and transport in rice, displays SeMet transport activity in yeast and *Xenopus* oocyte. The uptake rate of SeMet in the roots and its accumulation rate in the shoots of *nrt1.1b* mutant were significantly repressed. Conversely, the overexpression of *NRT1.1B* in rice significantly promoted SeMet translocation from roots to shoots, resulting in increased Se concentrations in shoots and rice grains. With vascular‐specific expression of *NRT1.1B*, the grain Se concentration was 1.83‐fold higher than that of wild type. These results strongly demonstrate that NRT1.1B holds great potential for the improvement of Se concentrations in grains by facilitating SeMet translocation, and the findings provide novel insight into breeding of Se‐enriched rice varieties.

## Introduction

Selenium (Se) is an essential trace element for humans and other animals that is critical for antioxidation by forming the active site of glutathione peroxidases as selenocysteine (SeCys) (Schwarz and Foltz, 1958; Rotruck *et al*., 1973; Steinbrenner and Sies, 2009). Se is also associated with multiple health benefits, such as enhancing immunity, delaying AIDS progression in HIV‐infected patients, reducing the incidence of cancers and maintaining male fertility (Clark *et al*., 1998; McKenzie *et al*., 1998; Foresta *et al*., 2002; Chantratita *et al*., 2004; Ryan‐Harshman and Aldoori, 2005; Shin *et al*., 2007). Many of these functions are mediated by selenoproteins, which contain Se in the form of SeCys. Sufficient Se intake supports maximal expression of selenoproteins (Xia *et al*., 2005), whereas inadequate dietary Se intake is closely associated with endemic diseases, such as Keshan disease, and Kashin–Beck disease (Tan *et al*., 2002). A recommended dietary allowance for health benefits is 50–60 μg/day for males and females (Institute of Medicine, 2000). However, the recommended daily intake is not achieved in the majority of European countries and in some parts of China (Rayman, 2004). Indeed, the majority of the world's population consumes less Se than the optimal amounts required for protection against cancer, cardiovascular diseases and other severe infectious diseases, and it is estimated that approximately one billion people worldwide suffer from Se deficiency (Combs, 2001; Haug *et al*., 2007).

Human Se is primarily acquired from plant foods, especially cereals, although milk, egg, meat and fish are also potential sources of Se (Rayman, 2012). Rice is a staple food for over half of the world's population. However, a survey of global rice samples revealed that approximately 75% of grains likely fail to provide 70% of the daily recommended Se intakes (Williams *et al*., 2009). Many factors affect Se concentration in grains, such as Se levels in soil, varietal differences, agronomic measures and the use of Se fertilizers (Eurola *et al*., 1991; Hartikainen, 2005; Hawkesford and Zhao, 2007; Lyons *et al*., 2005). Under soil Se‐deficient conditions, the use of Se fertilizers is often the only option to increase Se concentrations, and foliar spraying with Se fertilizers or supplying soils with fertilizers with Se can effectively increase Se concentration in grains (Deng *et al*., 2017; Eurola *et al*., 1991; Hartikainen, 2005; Li *et al*., 2010). However, foliar application of Se usually results in an uneven Se concentration in grains and increases the cost of Se‐enriched rice production; this technique is also difficult to perform under rainy or windy conditions. Thus, soil amendment of Se fertilizers is suggested as a feasible approach to increase grain Se concentration. However, Se added to soils is not efficiently taken up by plants. For example the total recovery (grain and straw) of applied Se was found to be only 20%–35%. The residual Se might be leached, volatilized by soil microbes, or retained in the soil as unavailable forms to plants (Broadley *et al*., 2010). In contrast, genetic biofortification, such as breeding Se‐enriched cultivars with high grain Se concentrations, provides a promising cost‐effective and sustainable approach to improve grain Se concentrations by enhancing the utilization efficiency of Se in soils.

Among the factors controlling Se accumulation in grains, Se uptake and transport are the most fundamental physiological processes. Selenate and selenite are the predominant forms of Se available to plants in soils. Because selenate is readily reduced to selenite under flooding conditions, rice plants growing in paddy soils mainly absorb selenite. Selenite naturally exists in diverse forms, such as H_2_SeO_3_, HSeO_3_^−^ and SeO_3_^2−^, in solutions (Läuchli, 1993), and H_2_SeO_3_ and HSeO_3_^−^ are taken up by rice roots through aquaporins and the phosphate transporter OsPT2 respectively (Li *et al*., 2008; Zhang *et al*., 2006a, 2014; Zhao *et al*., 2010). Although *OsPT2*‐overexpressing plants display a significantly enhanced root selenite uptake rate, the selenite absorbed is poorly translocated to shoots, thereby limiting the increase in Se concentration in grains (Zhang *et al*., 2014). Previous study suggested that a large proportion of the selenite absorbed by plant roots is transformed into organic Se compounds, such as SeMet (Kahakachchi *et al*., 2004; Li *et al*., 2008). Therefore, enhancing the efficiency of root‐to‐shoot SeMet translocation may increase grain Se concentration. However, the mechanism of SeMet transport in plants remains unclear. Plant peptide transporters (PTRs) transport a broad spectrum of substrates, such as nitrate, peptides, amino acids, dicarboxylates, glucosinolates, IAA and ABA (Leran *et al*., 2014). *NRT1.1B*, a member of the PTR family, encodes a protein containing a peptide‐transporter domain. We showed previously that NRT1.1B, predominantly expressed in the vascular tissues of roots, leaf sheaths, leaf blades and culms. Together with its plasma membrane localization, NRT1.1B was demonstrated to be involved in root‐to‐shoot nitrate transport (Hu *et al*., 2015). Here, we further reveal that NRT1.1B also mediates the root‐to‐shoot translocation of SeMet in rice. *NRT1.1B* overexpression significantly improved Se concentrations not only in shoots but also in grains. Our findings provide novel insights into breeding Se‐enriched rice varieties by facilitating SeMet translocation.

## Results

### SeMet is the dominant Se form in rice roots

**Figure 1:**
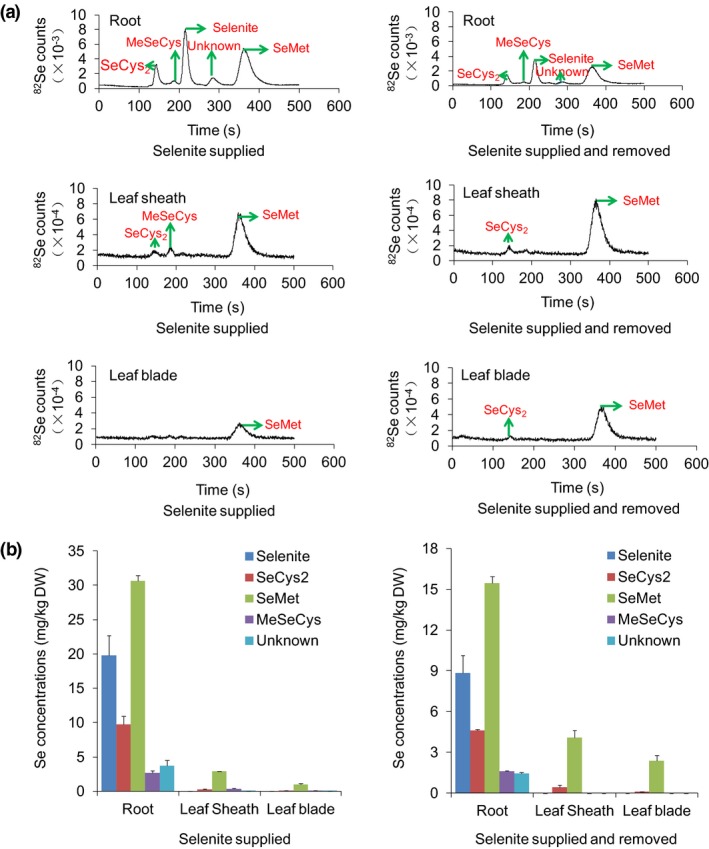
Assays of Se speciation in rice seedlings supplied with selenite. (a) Chromatograms of Se speciation based on HPLC‐ICP‐MS. (b) Se concentrations of different Se species in roots, leaf sheaths and leaf blades when supplied with selenite for 3 days and then cultured for another 3 days without Se. Values are the means ± SD (*n *=* *3).

As selenite is the dominant form of Se in paddy soil, we first examined Se forms in different organs of rice seedlings supplied with selenite for 3 days. Enzyme‐digested extracts of the roots, leaf sheaths and leaf blades were subjected to Se speciation analysis. The chromatogram of a mixed Se standard solution from HPLC‐ICP‐MS was provided as a reference (Figure S1). In the root extracts, selenite, SeMet, selenocystine (SeCys2), methylselenocysteine (MeSeCys) and unidentified Se forms were detected (Figure 1a). SeMet was the major Se form that constituted 46% of the total Se, followed by selenite and SeCys2, which accounted for 30% and 15% respectively; only trace amounts of MeSeCys (4%) and unidentified Se forms (6%) were detected. In the extracts of leaf sheaths and leaf blades, SeMet, SeCys2 and MeSeCys were found but not selenite (Figure 1a). SeMet was the dominant Se form, corresponding to 83% and 85% in leaf sheaths and leaf blades, respectively, and SeCys2 and MeSeCys accounted for 7% and 10% in leaf sheaths and 7% and 7% in leaf blades respectively.

After selenite treatment for 3 days, the rice seedlings were transferred back to nutrient solutions without Se for another 3 days, and we further investigated changes in the concentrations of various Se forms in different organs. Selenite and SeMet concentrations in the roots decreased by 2.24‐ and 1.98‐fold, respectively (Figure 1a,b), whereas the SeMet concentrations in leaf sheaths and leaf blades increased by 1.39‐ and 2.44‐fold respectively (Figure 1a,b). SeMet accounted for 91% and 96% in leaf sheaths and leaf blades respectively. These results revealed that most of the absorbed selenite was transformed to SeMet in the roots and then transported to the shoots.

Additionally, the root SeMet concentrations were 10.38‐ and 31.58‐fold higher than those in the leaf sheaths and leaf blades, respectively, after 3 days of selenite treatment, and the SeMet concentrations in the roots was 3.78‐ and 6.53‐fold higher than those in the leaf sheaths and leaf blades, respectively, when the seedlings were transferred back to nutrient solutions without Se for another 3 days (Figure 1b). This finding suggested that a large quantity of SeMet was retained in the roots and was not transported to the shoots.

### SeMet uptake is an energy‐dependent symport process

**Figure 2:**
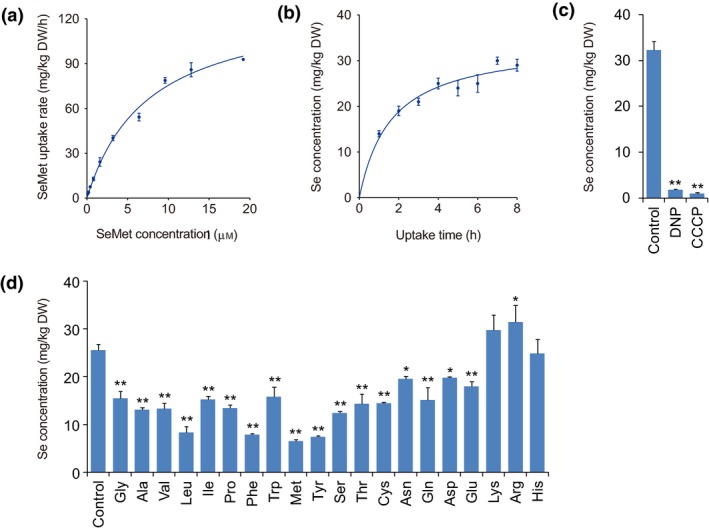
Assays of physiological characteristics of SeMet uptake by rice seedlings. (a) Concentration‐ and (b) time‐dependent SeMet uptake kinetics. (c) Effects of respiration inhibitors on SeMet uptake. (d) Competition assay of SeMet uptake using different amino acids. Values are the means ± SD (*n *=* *3). Asterisks indicate significant differences between control and treatments as evaluated by Student's *t*‐tests: *P <* *0.05 and **P <* *0.01.

To investigate whether SeMet is taken up via carriers, we performed concentration‐ and time‐dependent kinetic experiments of SeMet uptake with the rice seedlings. The results showed that the SeMet uptake rate increased markedly as the exogenous SeMet levels increased to 3.2 μm, then increased slowly and reached a plateau at high SeMet levels (Figure 2a). The SeMet uptake rate fit a Michaelis–Menten‐type correlation with the SeMet level. *V*_max_ and *K*_m_ were 132.30 ± 7.51 mg/kg dry weight·(DW) per h and 8.01 ± 1.05 μmol/kg DW, respectively, with *R* ^2^ of 0.99. Similarly, the Se concentration in the roots increased sharply over time and gradually reached a plateau at 4 h when the seedlings were supplied with SeMet (Figure 2b). This result indicated that SeMet uptake by rice roots exhibited the characteristic of saturation kinetics with time‐points. Thus, SeMet uptake was postulated to be a carrier‐mediated process supported by concentration‐ and time‐dependent saturation kinetics.

Carbonyl cyanide *m*‐chlorophenylhydrazone (CCCP) and 2, 4‐dinitrophenyl (DNP) are typical respiration inhibitors that collapse the proton‐motive force by allowing protons to freely transverse membranes. To determine whether SeMet uptake is an energy‐dependent symport process involving H^+^ transport, the effects of CCCP and DNP on SeMet uptake were investigated. The results indicated that CCCP and DNP decreased the Se concentration in roots by 94.5% and 97.0%, respectively, suggesting that CCCP and DNP largely inhibit SeMet uptake (Figure 2c). Therefore, SeMet uptake is an energy‐dependent symport process involving H^+^ transport.

### SeMet uptake is strongly inhibited by neutral amino acids

It has been postulated that SeMet, an analogue of Met, shares common transporters with other amino acids. To assess this hypothesis, we investigated the effects of amino acids on SeMet uptake by rice roots. Competitor amino acids were maintained at low concentrations (0.2 mm) to ensure that transport systems were active. It was found that most of the amino acids tested, except for the basic amino acids, lysine (Lys), arginine (Arg) and histidine (His), significantly inhibited SeMet uptake (Figure 2d). Neutral amino acids, namely, methionine (Met), tyrosine (Tyr), phenylalanine (Phe) and leucine (Leu), elicited the strongest inhibitory effects on SeMet uptake, by 74%, 71%, 69% and 67%, followed by serine (Ser), alanine (Ala), valine (Val), proline (Pro), threonine (Thr), cysteine (Cys), isoleucine (Ile), glutamine (Gln) and glycine (Gly) which inhibited SeMet uptake by 51%, 49%, 48%, 47%, 44%, 44%, 41%, 41% and 40% respectively. Tryptophan (Trp), asparagine (Asn) and the acidic amino acids, glutamic acid (Glu) and aspartic acid (Asp), elicited relatively slight inhibitory effects of 38%, 24%, 30% and 23% respectively. These results suggest that the carrier proteins responsible for the transport of neutral amino acids also mediate SeMet transport.

### *NRT1.1B* displays SeMet transport activity *in vitro*

**Figure 3:**
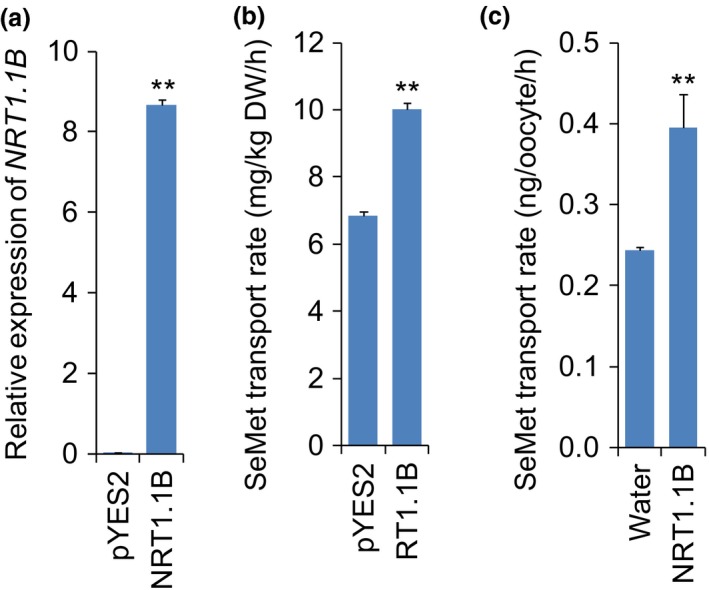
SeMet transport activity assays in yeast and oocyte. (a) Relative expression of *NRT1.1B* in yeast transformed with pYES2 empty vector and pYES2‐*NRT1.1B*. (b) SeMet transport rate in yeast transformed with pYES2 empty vector and pYES2‐*NRT1.1B*. (c) SeMet transport rate in the oocyte injected with *NRT1.1B* cRNA compared with that in the oocyte injected with water. Values are the means ± SD (*n *=* *5 for yeast and *n *=* *8 for oocyte). Asterisks indicate significant differences between pYES2 yeasts and pYES2‐*NRT1.1B* yeasts or between water and *NRT1.1B* cRNA injected oocytes as evaluated by Student’s t‐tests: *P <* *0.05 and **P <* *0.01.

To investigate whether NRT1.1B is involved in SeMet transport, SeMet transport activity of NRT1.1B was examined *in vitro*. Yeast strains expressing a full‐length *NRT1.1B* cDNA were constructed and incubated in liquid medium containing SeMet. According to the results of qRT‐PCR, expression of *NRT1.1B* was substantially increased in the *NRT1.1B*‐transgenic strain relative to the control strain carrying empty vector (Figure 3a). The SeMet transport rate in the *NRT1.1B*‐transgenic strain was significantly higher than that in the control strain (Figure 3b). The SeMet transport activity of NRT1.1B was also evaluated in *Xenopus* oocytes, and uptake measurement showed that the SeMet transport rate in the oocytes injected with *NRT1.1B* cRNA was significantly higher than that in the oocytes injected with water (Figure 3c). These results strongly demonstrated that NRT1.1B has a transport activity for SeMet.

### *nrt1.1b* mutant displays defects in SeMet uptake and transport

**Figure 4:**
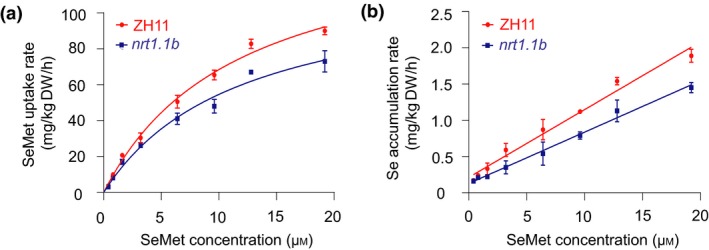
(a) SeMet uptake was repressed in *nrt1.1b* mutant. (a) Concentration‐dependent kinetics of SeMet uptake by roots and (b) accumulation in shoots between wild type (ZH11) and *nrt1.1b* mutant. Values are the means ± SD (*n *=* *3).

To determine the potential function of NRT1.1B in SeMet uptake and transport in rice plant, we further characterized its loss‐of‐function mutant *nrt1.1b* (Hu *et al*., 2015). Concentration‐dependent SeMet uptake kinetics showed that with increasing exogenous SeMet concentrations, the uptake rate of SeMet in wild type and *nrt1.1b* mutant fit well into a Michaelis–Menten‐type correlation (Figure 4a). The uptake rate of SeMet in wild type was significantly higher than that in *nrt1.1b* mutant at different SeMet levels. *V*_max_ and *K*_m_ were 148.42 ± 7.55 mg/kg DW/h and 11.69 ± 1.16 μmol/kg DW for wild type, respectively, and 119.20 ± 7.55 mg/kg DW/h and 11.85 ± 1.77 μmol/kg DW for *nrt1.1b* mutant respectively. *R* ^2^ was 0.99 and 0.98 for wild type and *nrt1.1b* respectively. Although wild type and *nrt1.1b* mutant displayed the same affinity for SeMet, the *V*_max_ of wild type was significantly higher than that of *nrt1.1b*, indicating that the mutation of *NRT1.1B* may cause a decrease in *V*_max_ and consequently result in a defect in SeMet uptake.

Concentration‐dependent SeMet accumulation kinetics revealed that the Se accumulation rate in the shoots of wild type and *nrt1.1b* increased linearly, with linear correlations with exogenous SeMet concentrations (Figure 4b). For wild type, the linear regression equation and *R* ^2^ were *Y* = 93.274*X* + 213.14 and 0.98 respectively; for *nrt1.1b*,the results were *Y* = 68.526*X* + 127.91 and 0.99 respectively. As the Se accumulation rates within 2 h in the shoots of the wild type were significantly higher than those of the *nrt1.1b* mutant at different SeMet levels, NRT1.1B possibly mediates root‐to‐shoot transport of SeMet.

### *NRT1.1B* overexpression increases root‐to‐shoot translocation of SeMet

**Figure 5:**
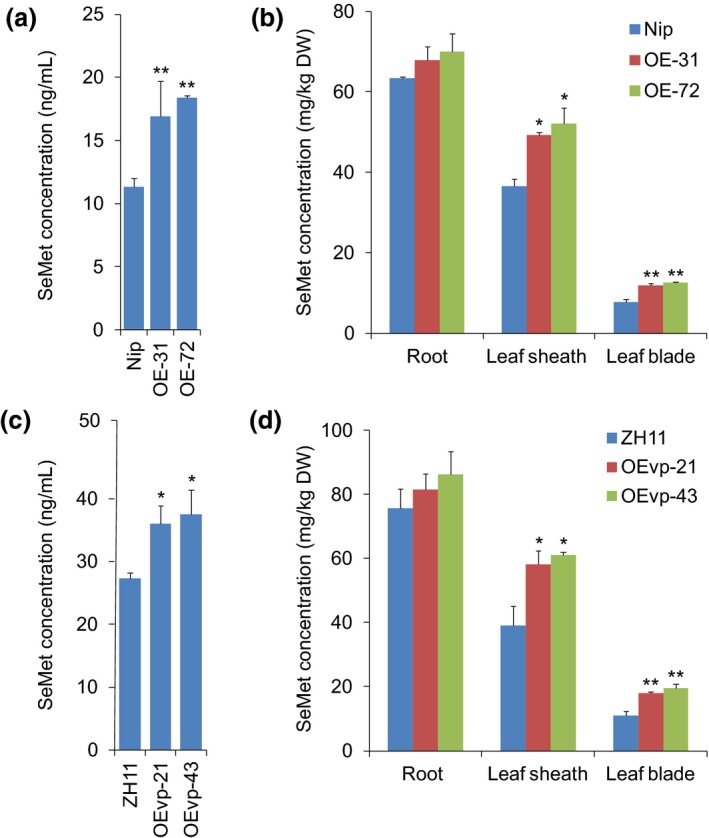
Overexpression of *NRT1.1B* enhances SeMet transport in rice seedlings. SeMet concentrations in xylem sap collected from different *NRT1.1B‐*overexpressing lines (a) OE‐31/OE‐72 and (c) OEvp‐21/OEvp‐43 supplied with 50 μm SeMet for 6 h and then transferred back to nutrient solutions without Se for 1 day. SeMet concentrations in rice roots, leaf sheaths and leaf blades of overexpressing lines (b) OE‐31/OE‐72 and (d) OEvp‐21/OEvp‐43 supplied with SeMet for 3 h and then transferred back to nutrient solutions without Se for 3 days. The wild type controls for OE‐31/OE‐72 and OEvp‐21/OEvp‐43 are Nipponbare (Nip) and Zhonghua 11 (ZH11) respectively. Values are the means ± SD (*n *=* *3). Asterisks indicate significant differences between wild‐type plants and transgenic lines as evaluated by Student's *t*‐tests: *P <* *0.05 and **P <* *0.01.

To investigate whether NRT1.1B increases SeMet translocation from roots to shoots, we performed SeMet transport assays in wild type (Nip) and *NRT1.1B‐*overexpressing plants exposed to SeMet. Two lines generated using the rice *ACTIN1* promoter, OE‐31 and OE‐72, with significantly increased expression of *NRT1.1B* were selected for further study (Figure S2a). SeMet concentration in xylem sap was used as an indicator of the capacity of SeMet translocation from roots to shoots, and the results showed that the SeMet concentration in xylem sap collected from OE‐31 and OE‐72 was 1.50‐ and 1.65‐fold higher, respectively, than that from the wild‐type plants (Figure 5a). In addition, the ratio of SeMet content between the shoot and root could represent the activity of long‐distance SeMet transport (Zayed *et al*., 1998), and our results showed that the ratios of SeMet content of leaf blade/root, leaf sheath/root and shoot/root OE‐31 and OE‐72 were significantly higher than those of wild type (Figure S3a). Thus, overexpression of *NRT1.1B* significantly enhanced SeMet translocation from roots to shoots. Moreover, compared with wild type, the SeMet concentration in the leaf sheaths and leaf blades was increased by 1.35‐ and 1.43‐fold, respectively, for OE‐31 and by 1.53‐ and 1.61‐fold, respectively, for OE‐72 (Figure 5b). These findings suggest that the increased activity of SeMet transport is critical for improving the Se accumulation in shoots.

The rice *ACTIN1* promoter used to generate overexpressing lines OE‐31 and OE‐72 is constitutively expressed in most tissues. However, this expression pattern might not be optimal for enhancing SeMet transport compared with that mainly expressed in the vascular tissues. Our previous study indicated that the *gdcs*P promoter is specifically expressed in vascular tissues, with most abundant expression in the roots, followed by the spike stalks, stems, leaf blades and leaf sheaths (Chen *et al*., 2001). We thereby generated *NRT1.1B‐*overexpressing plants driven by *gdcs*P, and two lines, OEvp‐21 and OEvp‐43, with significantly increased expression of *NRT1.1B* were selected for further study (Figure S2a). We found the SeMet concentration in xylem sap collected from OEvp‐21 and OEvp‐43 was 2.05‐ and 2.23‐fold higher than that in wild‐type plants (ZH11) respectively (Figure 5c). In addition, the leaf blade/root, leaf sheath/root and shoot/root SeMet content ratios of OEvp‐21 and OEvp‐43 were also significantly higher than those of wild type (ZH11) (Figure S3b). Compared with wild‐type plants (ZH11), the SeMet concentrations in leaf sheath and leaf blade were increased by 1.49‐ and 1.63‐fold, respectively, for OEvp‐21 and by 1.57‐ and 1.77‐fold, respectively, for OEvp‐43 (Figure 5d). These results collectively suggest that vascular‐specific expression of *NRT1.1B* dramatically increased SeMet translocation from roots to shoots.

### *NRT1*.*1B* overexpression increases Se concentrations when supplied with selenite

**Figure 6:**
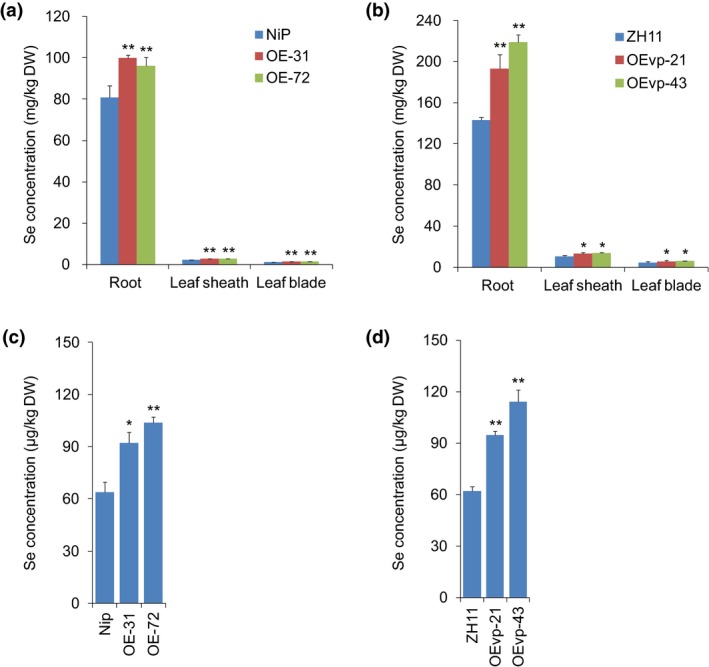
Overexpression of *NRT1.1B* increases Se concentrations in transgenic rice. Se concentration assays in the seedlings of *NRT1.1B* overexpressing lines (a) OE‐31/OE‐72 and (b) OEvp‐21/OEvp‐4 when supplied with selenite. Se concentration assays in grains of *NRT1.1B* overexpressing lines (c) OE‐31/OE‐72 and (d) OEvp‐21/OEvp‐43. Values are the means ± SD (*n *=* *3). Asterisks indicate significant differences between wild‐type plants and transgenic lines as evaluated by Student's *t*‐tests: *P <* *0.05 and **P <* *0.01.

Because selenite is the dominant Se form in paddy soils, we further investigated whether *NRT1.1B* overexpression would increase Se concentrations when rice seedlings were exposed to selenite. As expected, Se concentrations in the roots, leaf sheaths and leaf blades of OE‐31 were significantly increased by 1.24‐, 1.24‐ and 1.21‐fold, respectively, and those of OE‐72 were 1.19‐, 1.32‐ and 1.31‐fold, respectively, compared with wild type (Nip) (Figure 6a). Similarly, compared with wild type (ZH11), Se concentrations in the roots, leaf sheaths and leaf blades of OEvp‐21 were increased by 1.35‐, 1.25‐ and 1.32‐fold, and those of OEvp‐43 were increased by 1.53‐, 1.31‐ and 1.37‐fold (Figure 6b). These results collectively suggested that *NRT1.1B* overexpression facilitates more Se translocation from roots to shoots, thereby resulting in increased Se concentrations in leaf sheaths and leaf blades of rice seedlings.

Previous study revealed that OsPT2 and OsNip2;1 are involved in selenite uptake in rice plant (Zhang *et al*., 2014; Zhao *et al*., 2010), thus we examined the expression levels of *OsPT2* and *OsNip2;1* in the roots of *NRT1.1B*‐overexpressing lines. Interestingly, expression of *OsPT2* and *OsNip2;1* was significantly increased in the roots of OE‐31 and OE‐72, and that of *OsPT2* was also significantly increased in the roots of OEvp‐21 and OEvp‐43, which may also promote selenite uptake in paddy soils (Figure S4a,b).

### *NRT1.1B* overexpression improves Se concentrations in rice grains

To further investigate whether NRT1.1B increases Se concentrations in rice grains, we examined Se concentrations in the grains of wild type and *NRT1.1B‐*overexpressing plants grown in the field. Expression of *NRT1.1B* was significantly increased by 29.73‐ and 39.30‐fold in the shoots of OE‐31 and OE‐72, respectively, compared with that of wild type (Nip), and it was increased by 1.82‐ and 7.88‐fold in the shoots of OEvp‐21 or OEvp‐43, respectively, compared with that of wild type (ZH11) (Figure S2b). As expected, Se concentrations were significantly increased in the grains of the overexpressing lines, reaching 1.25‐ and 1.43‐fold in OE‐31 and OE‐72, respectively, compared with wild type (Nip) (Figure 6c). Notably, the grain Se concentrations of OEvp‐21 and OEvp‐43 were increased by 1.53‐ and 1.83‐fold compared with wild type (ZH11) (Figure 6d), even though the increase in *NRT1.1B* expression in OEvp‐21 and OEvp‐43 was much lower than that in OE‐31 and OE‐72 (Figure S2b), displaying a more significant effect in improving grain Se concentrations. In addition, the plant yield of OEvp‐21 and OEvp‐43 was a little higher than that of wild type, though the difference was not significant (Figure S5a). The grain weight also exhibited no significant difference between overexpressing line OEvp‐21 or OEvp‐43 and wild type (Figure S5b). These results indicate that NRT1.1B holds great potential to increase grain Se concentrations with no repercussions to grain yield.

## Discussion

Se naturally occurs in soils as selenide, elemental Se, thioselenate, selenite and selenate (Läuchli, 1993). Selenate and selenite are the two dominant forms of Se available to plants, and redox potential and pH can greatly affect the forms of Se present in soils. Thermodynamic calculations show that selenate should be the predominant form in alkaline and well‐oxidized soils (pE + pH> 15), whereas selenite is the form in reduced soils with pH from acidic to neutral (7.5 <  pE + pH < 15) (Elrashidi *et al*., 1987; Li *et al*., 2008). Thus, selenite is the dominant Se form available to rice roots in paddy soils under flooding conditions. Our Se speciation analysis revealed that selenite was only present in roots and not in shoots (Figure 1), indicating that the selenite absorbed by rice roots was predominantly transformed into SeMet and was further translocated to shoots in the form of organic Se. We showed previously that selenite can be taken up through OsPT2, a phosphate transporter responsible for transporting inorganic phosphate (Pi) from roots to shoots (Ai *et al*., 2009; Zhang *et al*., 2014). However, a perplexing issue was encountered in the present study, namely, why selenite in roots was not directly transported to shoots via OsPT2. Actually, selenite is non‐enzymatically quickly reduced to GSH‐SeO_3_^−^ and GS‐Se‐GS in the cytoplasm and then reduced to GSH‐Se^−^ once it enters root epidermal cells (Anderson, 1993; Anderson and Scarf, 1983). GSH‐Se^−^ reacts with *O*‐acetyl serine to form SeCys in plastids (Ng and Anderson, 1978a,b). Most of SeCys is transformed into SeMet and a small portion of SeCys is converted into MeSeCys (Neuhierl and Bock, 1996). Selenite metabolism in rice roots was further demonstrated via detection of a large quantity of SeMet and a trace amount of SeCys2 and MeSeCys in roots. This finding further revealed that most selenite was transformed into organic Se, resulting in a high concentration ratio of Pi to selenite in the cytoplasm of root cells. Moreover, OsPT2 is postulated to have a much higher affinity for Pi than for selenite. Thus, selenite was not found to be translocated from roots to shoots via OsPT2.

When rice seedlings were supplied with selenite, SeMet was the predominant form of Se in roots (Figure 1a). A previous study has indicated that the ratio of shoot Se to root Se ranges from 0.6 to 1.0 for plants supplied with SeMet, thereby suggesting that SeMet is not efficiently transported from roots to shoots (Zayed *et al*., 1998). In the present work, SeMet concentrations in roots of wild type (Nip) were 1.74‐ and 8.17‐fold higher than those in leaf sheaths and leaf blades, respectively, when wild‐type seedlings were supplied with SeMet for 3 h and then transferred back to nutrient solutions without Se for 3 days (Figure 5b). These results further demonstrated that a large quantity of SeMet was not transported to shoots but instead was retained in the roots. This finding may explain why Se was not readily translocated from roots to shoots when seedlings were supplied with selenite because a large proportion of absorbed selenite was predominantly transformed to SeMet in the roots. Thus, improving the capacity of SeMet transport from roots to shoots is a key step for enhancing Se transport when rice seedlings are supplied with selenite.

Concentration‐ and time‐dependent uptake kinetics indicated that SeMet uptake exhibited saturation characteristics (Figure 2a,b). Moreover, SeMet uptake was largely inhibited by respiration inhibitors, such as CCCP and DNP (Figure 2c), a finding that is also supported by a previous study (Abrams *et al*., 1990). Therefore, SeMet uptake is an active process requiring a selective binding site and metabolic energy as a driving force. In addition, we found that SeMet uptake was remarkably inhibited by neutral amino acids, such as Met, Tyr, Phe, Leu, Ser, Ala, Val, Pro, Thr, Cys and Gln (Figure 2d), suggesting that SeMet shares common transporters with neutral amino acids. The molecular functions of several amino acid transporters (AATs) are characterized in *Arabidopsis* and rice. For instance AtAAPs transport neutral and charged amino acids (Fischer *et al*., 2002; Lee *et al*., 2007), and AtANTs are involved in transporting aromatic and neutral amino acids (Hirner *et al*., 2006); OsAAPs can transport neutral, acidic and basic amino acids (Lu *et al*., 2018; Peng *et al*., 2014; Taylor *et al*., 2015). These findings suggest that different types of AATs are able to transport a broad spectrum of neutral amino acids. In addition, one type of AATs can transport a broad spectrum of neutral amino acids, whereas one type of neutral amino acids can also be transported by different types of AATs. Thus, SeMet may be nonspecifically transported via a wide range of AATs responsible for transporting neutral amino acids. However, as neutral amino acids, such as Ala, Ser, Met, Gln and Asn, can greatly inhibit NO_3_^–^ uptake (Aslam *et al*., 2001; Dluzniewska *et al*., 2006; Gessler *et al*., 1998; Miller *et al*., 2008; Muller and Touraine, 1992), enhancing SeMet transport by overexpressing AAT genes may nonspecifically increase neutral amino acid accumulation, possibly resulting in the repression of nitrogen utilization. Therefore, it would be desirable to seek specific transporters responsible for transporting SeMet.

Peptide transporters can also transport amino acids in addition to a broad spectrum of other substrates such as nitrate, peptides, dicarboxylates, glucosinolates, IAA and ABA in plants (Leran *et al*., 2014). For example BnNRT1;2 exhibits a similar transport activity for nitrate and His (Zhou *et al*., 1998). AtPTR1 is a high‐affinity peptide transporter that can also transport His (Dietrich *et al*., 2004). Because peptide transporters such as BnNRT1;2 and AtPTR1 exhibit transport activity for only a few amino acids, such as His (Dietrich *et al*., 2004; Zhou *et al*., 1998), these transporters have been postulated to have a higher selectivity for transporting amino acids than do AATs. Our previous study showed that NRT1.1B, a member of the rice PTR family, is involved in root‐to‐shoot nitrate translocation (Hu *et al*., 2015). Therefore, whether NRT1.1B also functions in the transport of SeMet is an extremely intriguing question. As expected, NRT1.1B showed transport activity for SeMet, as demonstrated in yeast and in oocytes. Moreover, the SeMet uptake rate of *nrt1.1b* mutant was significantly lower than that of wild‐type rice. Further studies revealed that overexpression of *NRT1.1B* in rice significantly increased SeMet concentration in the xylem sap and in shoots when plants were supplied with SeMet (Figure 5a–d). In addition, the ratio of the SeMet content between shoots and roots in *NRT1.1B*‐overexpressing lines was significantly higher than that in wild type (Figure S3a,b). These findings collectively confirm that NRT1.1B possesses SeMet transport activity and can mediate SeMet translocation from roots to shoots in rice. *NRT1.1B* is predominantly expressed in the vascular tissues of root, leaf sheath, leaf blade and culm (Hu *et al*., 2015), further supporting its role in transporting SeMet from roots to shoots.

Met, an analogue of SeMet, can inhibit SeMet uptake by 74%, suggesting that Met shares common transporters with SeMet (Figure 2d). Thus, overexpression of *NRT1.1B* may also stimulate Met translocation from roots to shoots. However, peptide transporters have a higher selectivity for transporting amino acids compared with AATs (Dietrich *et al*., 2004; Zhou *et al*., 1998). Thus, even though other neutral amino acids such as Tyr, Phe and Leu elicit the strongest inhibitory effects on SeMet uptake, but further investigation is required to determine whether overexpression of NRT1.1B may increase the translocation of these amino acids. Additionally, although SeMet and nitrate are common substrates of NRT1.1B, SeMet is significantly different from nitrate in both physical and chemical characteristics. It is therefore imaginable that SeMet and nitrate may use different binding sites of NRT1.1B. A previous study found that nitrate up‐regulated expression of *NRT1.1B* (Hu *et al*., 2015), thus, SeMet uptake and transport may be enhanced by nitrate through induction of *NRT1.1B* expression.

In vascular tissues of rice leaf blades, xylem and phloem occur in close proximity to each other, and the metaphloem sieve tubes in rice leaf veins are either in direct contact with or in close proximity to metaxylem vessels (Botha *et al*., 2008). Thus, SeMet exchange between xylem and phloem may occur after SeMet is translocated from the roots to leaf blades. Once SeMet reaches the xylem parenchyma, it may be transported through the phloem parenchyma and then into companion cell‐sieve tube complexes. Because NRT1.1B is highly expressed in leaf blade vascular tissues (Hu *et al*., 2015), SeMet can be transferred by NRT1.1B from the xylem to the phloem in leaf veins to ensure efficient remobilization of SeMet to seeds.

Our previous study found that Se concentrations in rice grains correlated positively with those in shoots (Zhang *et al*., 2006b). Because overexpression of *NRT1.1B* enhanced Se concentrations in shoots by increasing SeMet translocation from roots to shoots when supplied with selenite (Figure 6a,b), it was postulated that overexpression of this gene may also increase Se concentrations in grains. As expected, Se concentrations in the grains of the *NRT1.1B*‐overexpressing transgenic lines OE‐31 and OE‐72 were significantly increased compared with those in wild type (Nip) (Figure 6c). Interestingly, although vascular‐specific overexpression of *NRT1.1B* did not significantly increase grain yield, the Se concentration in the grains of OEvp‐43 was increased up to 1.83‐fold than that of wild‐type plants (ZH11), providing a refined strategy for manipulating *NRT1.1B* expression to improve Se accumulation in grains (Figure 6d). SeMet has been reported as the main form of organic Se in rice grains (Fang *et al*., 2009; Li *et al*., 2010; Sun *et al*., 2010; Zhao *et al*., 2011), and as such, overexpression of *NRT1.1B* may increase SeMet translocation to grains. These results strongly demonstrated that overexpression of *NRT1.1B* could improve Se concentrations in grains by facilitating SeMet translocation. This study not only enhances our understanding of Se translocation from roots to shoots in rice, but also provides novel insight into breeding Se‐enriched rice varieties by facilitating SeMet translocation.

## Experimental procedures

### Plant materials and growth conditions

Rice (*Oryza sativa* L.) wild‐type Zhonghua 11 (ZH11), its *nrt1.1b* mutant and vascular‐specifically *NRT1.1B‐*overexpressing lines OEvp‐21 and OEvp‐43, wild‐type Nipponbare (Nip) and its *NRT1.1B*‐overexpressing lines OE‐31 and OE‐72 were used in this study. The *NRT1.1B‐*overexpressing lines OE‐31 and OE‐72 were generated using rice *ACTIN1* promoter, which is constitutively expressed in most tissues. The *NRT1.1B*‐overexpressing lines OEvp‐21 and OEvp‐43 were generated using *gdcs*P, a vascular‐specific promoter isolated from *Faveria anomala* (Chen *et al*., 2001). The *nrt1.1b* mutant carries a T‐DNA insertion in the intron and has defects in both nitrate uptake and nitrate root‐to‐shoot transportation (Hu *et al*., 2015). Seeds were surface‐sterilized in 1% NaClO solution for 10 min, and then germinated in an incubator at 35°C. The young seedlings were transferred to Kimura B nutrient solution. The nutrient solutions were renewed every 3 days. pH was adjusted to 5.5 every day. The seedlings were cultured in a controlled growth chamber with a diurnal cycle of 14 h light at 25 °C and 10 h dark at 18 °C. The light intensity was 300 μmol·photons/m^2^/s. The air humidity was controlled at 67%. Rice seedlings were continuously cultured for 3–4 weeks for experiments (Zhang *et al*., 2014).

### Enzyme hydrolysis

Ground samples (0.1 g) were placed into 10 mL centrifuge tubes, and protease K (20 mg), lipase VII (10 mg) and pH 7.5 Tris‐HCl were added up to a final volume of 5 mL. The tubes were gently shaken at 37 °C with 60 r.p.m. for 22 h in darkness, and then centrifuged at 1800 ***g*** for 30 min. The supernatants were collected, filtered through 0.45 μm filter and stored at −20 °C for Se speciation analysis (Sun *et al*., 2010).

### Se speciation analysis of rice seedlings cultured in selenite solution

About 18 days of rice seedlings were transferred to Kimura B nutrient solution containing 2 μm Na_2_SeO_3_, and the rice seedlings were harvested for Se speciation analysis after 3 days. The other seedlings were transferred back to nutrient solutions without Se for another 3 days before being harvested for Se speciation analysis to investigate changes in the concentrations of Se forms. High‐performance liquid chromatography (HPLC, Agilent Technologies LC1100 series) coupled with inductively coupled plasma mass spectrometry (ICP‐MS, 7500ce, Agilent Technologies) was used for the Se speciation analysis. The different Se species were separated by an anion‐exchange column (PRP X‐100, 4.1 mm×250 mm, 10 μm) and a precolumn (Dionex AG14). The injection volume of individual sample was 50 μL. The mobile phase contained 5 m ammonium citrate and 2% methanol (pH 4.3) at a flow rate of 1 mL/min was used. The ICP‐MS operating conditions were as follows: RF power, 1400 W; sample depth, 8 mm from the load coil; Ar auxiliary gas flow rate, 0.8 L/min; spray chamber temperature, 2 °C. Peaks were identified by comparison with the retention times of the standard compounds. The Se standard sample was a mixture of 50 μg/L selenite, SeCys2, SeMet and MeSeCys, respectively, which were purchased from Sigma. Se species were identified by the retention times and quantified by peak area measurements of the chromatographic signals by monitoring the isotope ^82^Se (Sun *et al*., 2010).

### Kinetics assay of SeMet uptake and accumulation

The roots of the rice seedlings (ZH11 and its *nrt1.1b* mutant) were transferred to absorption solution containing 5 mm 2‐morpholinoethanesulphonic acid (MES), 0.5 mm Ca(NO_3_)_2_ and SeMet at concentrations of 0, 0.4, 0.8, 1.6, 3.2, 6.4, 9.6, 12.8 or 19.2 μm (pH 5.0) for 2 h. After the termination of SeMet uptake, the roots were rinsed to remove the Se adsorbed onto the root surfaces. The roots and shoots were then separated for Se concentration analysis. Similarly, the roots of rice seedlings (ZH11) were placed in absorption solution containing 5 mm MES, 0.5 mm Ca(NO_3_)_2_ and 2 μm SeMet for 1, 2, 3, 4, 5, 6, 7 or 8 h. The roots were separated for Se concentration analysis (Zhang *et al*., 2014).

### Assay of SeMet uptake affected by respiration inhibitors

The roots of rice seedlings were placed in absorption solution containing 5 mm MES, 0.5 mm Ca(NO_3_)_2_, 2 μm SeMet with and without 1 μm CCCP or 20 μm DNP, respectively, for 2 h. After the termination of SeMet uptake, the roots were rinsed and excised from the base of the shoots for Se concentration analysis (Zhang *et al*., 2014).

### Competitive assay of SeMet uptake with amino acids

The roots of rice seedlings were placed in absorption solution containing 5 mm MES, 0.5 mm Ca(NO_3_)_2_, and 1 μm SeMet with 0.2 mm amino acid, including Gly, Ala, Val, Leu, Ile, Pro, Phe, Trp, Met, Tyr, Ser, Thr, Cys, Asn, Gln, Asp, Glu, Lys, Arg or His, respectively, for 2 h. After the termination of SeMet uptake, the roots were rinsed and excised from the base of the shoots for Se concentration analysis.

### Determination of Se concentration

Dried and homogenized samples were weighed and put into 100 mL digestion tubes. Then 5 mL of an acid mixture of 4 mL HNO_3_ and 1 mL HClO_4_ were added. The samples were pre‐digested overnight at room temperature, and then digested at 150 °C completely in a digestion oven. The digests were diluted with millipore water to a final volume of 25 mL. Total Se in the digested samples was determined by ICP‐MS (Zhang *et al*., 2014).

### Construction of rice plants overexpressing *NRT1.1B*

The CDS of *NRT1.1B* (*japonica*) was amplified and cloned into the binary vector pCAMBIA2300‐CaMV 35S or pCAMBIA 2300‐*gdcs*P between restriction sites *Sma*I and *Xba*I to generate pCAMBIA2300‐35S: *NRT1.1B* or pCAMBIA2300‐*gdcs*P: *NRT1.1B*‐overexpressing vectors respectively. The resulting vectors and the empty vectors were introduced into *Agrobacterium* strain AGL1 respectively. Wild type (Nip or ZH11) were used as the recipients for *Agrobacterium*‐mediated transformation to generate the transgenic rice as described previously (Liu *et al*., 2007). T_0_ generation of transgenic plants were further identified using PCR amplification of NPTII with the genome DNA. The relative expression of *NRT1.1B* was determined using real‐time quantitative reverse transcription polymerase chain reaction (qRT‐PCR) in *NRT1.1B*‐overexpressing lines and wild type (Hu *et al*., 2015).

### SeMet uptake assay in yeast

The full‐length *NRT1.1B* cDNA was subcloned into pYES2 between the restriction sites *Bam*HI and *Eco*RI to generate pYES2‐*NRT1.1B* vector. The resulting vector and the empty vector were introduced into yeast strain 135‐A‐8 (*MATα ptr2‐his3∆1 leu2∆0 lys2∆0 ura3∆0*). Relative expression was quantitatively analysed using qRT‐PCR to guarantee higher expression of *NRT1.1B* in *NRT1.1B*‐transgenic strain compared to the empty vector transgenic strain. Transformed yeast cells carrying pYES2‐NRT1.1B or pYES2 were incubated in the YPD liquid medium at 30 °C with shaking at 250 r.p.m. until OD_600_ reached 0.6 (about 16–20 h) respectively. The yeast cells were collected by centrifugation at 700 ***g***, and then cultured in YPD liquid medium containing 2 μm SeMet at 30 °C with shaking at 250 r.p.m. for 3 h. The yeast cells were collected by centrifugation and rinsed with sterile H_2_O five times to remove the rest SeMet. Finally, the rinsed yeast cells were weighed and digested for Se concentration analysis (Hu *et al*., 2015).

### SeMet uptake assay in *Xenopus laevis* oocytes

The coding region of *NRT1.1B* (*japonica*) was amplified and cloned into the *Xenopus laevis* oocyte expression vector pCS2+ between the restriction sites *Bam*HI and *Eco*RI and then linearized with *Apa*I. Capped mRNA was synthesized *in vitro* using the mMESSAGE mMACHINE kit (Ambion, AM1340) according to the manufacturer's protocol. *X. laevis* oocytes at stage V–VI were injected with 46 ng of *NRT1.1B* cRNA in 46 nL nuclease‐free water. After injection, the oocytes were cultured in ND96 medium containing 200 μm SeMet for 6 h and used for SeMet uptake assays (Hu *et al*., 2015).

### Collection and SeMet determination of xylem sap

Rice seedlings of different genotypes were cultured in Kimura B nutrient solution for 3 weeks as described above. The seedlings were then placed in absorption solution containing 5 mm MES, 0.5 mm Ca(NO_3_)_2_ and 50 μm SeMet for 6 h, and then transferred back to nutrient solutions without Se for 1 day. The rice seedlings of different genotypes were decapitated at the base of the shoots with a sharp blade. To avoid contamination with cellular constituents from the cut, the first drops of xylem sap exuded from the cut surface of the decapitated shoots were discarded. The xylem sap was collected manually with micropipettes for 1.5 h after decapitation and temporarily stored at −20 °C. The sap samples were enzymatically hydrolysed as described above, and the SeMet concentration of the sap was analysed by HPLC‐ICP‐MS (Sun *et al*., 2010).

### Assay of SeMet transport

The rice seedlings of different genotypes were placed in absorption solutions containing 5 mm MES, 0.5 mm Ca(NO_3_)_2_ and 6 μm SeMet for 3 h, and then transferred back to nutrient solutions without Se for 3 days. SeMet concentration was determined in the roots, leaf sheaths and leaf blades, respectively, by HPLC‐ICP‐MS (Sun *et al*., 2010), and then SeMet content ratios of leaf blades to roots, leaf sheaths to roots and shoots to roots of *NRT1.1B‐*overexpressing lines OE‐31/OE‐72 and OEvp‐21/OEvp‐43 were calculated.

### Assay of Se concentration cultured in selenite solution

The rice seedlings of different genotypes were cultured in Kimura B nutrient solution containing 2 μm Na_2_SeO_3_ for 3 days, and then harvested. Se concentration was determined in the roots, leaf sheaths and leaf blades, respectively, by ICP‐MS (Zhang *et al*., 2014).

### Expression assay of *OsPT2, OsNIP2.1* and *NRT1.1B* in *NRT1.1B‐*overexpressing lines

Rice seedlings of different genotypes were cultured in Kimura B nutrient solution for 2 weeks. The roots were harvested for total RNA extraction to determine the expression of *OsPT2* and *OsNIP2.1* in the *NRT1.1B*‐overexpressing lines using qRT‐PCR. The primers are as following: *OsPT2* forward primer, cacaaacttcctcggtatgct; *OsPT2* reverse primer, gaaaccccacaaatccacaac; *OsNIP2.1* forward primer, ggggcaatttcaggtggatcg; *OsNIP2.1* reverse primer, ttctgggaggagccttcctt. Similarly, the roots and shoots were harvested to determine the expression of *NRT1.1B*. Forward primer, ggcaggctcgactacttcta; reverse primer, aggcgcttctccttgtagac (Hu *et al*., 2015).

### Field experiment

The rice seedlings of different genotypes were grown in the field in Lingshui County of Hainan Province, China. The spacing between plants was 20 cm. The plot size for each genotype was 10 m^2^, with three replicates. The concentrations of organic matter, available N, P and K in the soils were 22 415, 98.74, 41.42 and 144.05 mg/kg respectively. The concentrations of total Se and available Se were 0.530 mg/kg and 5.78 μg/kg respectively. Available N was determined by diffusion dish method. Soil samples were hydrolysed to NH_3_ by 1.2 m NaOH solution and continuously absorbed by 2% H_3_BO_3_, and then titrated by 0.01 m HCl solution. Available P was extracted by an acid mixture of 0.05 m HCl and 0.025 mm H_2_SO_4_ and determined by molybdenum antimony colorimetric method. Available K was extracted by 1 m neutral NH_4_OAC and determined by flame photometer method. Total Se in soil samples was extracted and determined following Se concentration determination of plant samples as described above. Available Se was extracted by 0.05 m KH_2_PO_4_ and 0.05 m K_2_HPO_4_ and determined as described above. The soil type was clay with pH 5.6. The cultural management practices were the same for different genotypes. Upon ripening, rice grains were harvested and dried at 50 °C in an oven, and then the Se concentration was determined by ICP‐MS (Zhang *et al*., 2014). In addition, rice grains of overexpressing lines OEvp‐21 and OEvp‐43 from a single plant were collected for measurements of grain yield per plant. Randomly picked filled grains were used for 1000‐grain weight measurements. Ten and five biological replications were performed for grain yield per plant and 1000‐grain weight respectively (Hu *et al*., 2015).

### Statistical analysis

One‐way analysis of variance (ANOVA) was performed using SPSS 13.0 for Windows (SPSS Inc., Chicago, IL) to determine the significant differences (*P *<* *0.05 and *P *<* *0.01) between control and treatments. Statistical differences were assessed by Student's *t*‐test (Zhang *et al*., 2014).

## Author contributions

L. Zhang., C. Chu, Y. Li and H. Ling designed research. B. Hu., K. Deng., X. Gao., G Sun., Z. Zhang., P. Li, W. Wang., H. Li., Z. Zhang., L. Li., Z. Fu., J. Yang., S. Gao. and F. Yu performed research; L. Zhang., K. Deng., X. Gao and B. Hu analysed the data; and L. Zhang wrote the paper.

## Conflict of interest

The authors declare no competing financial interests.

## Supporting information


**Figure S1** Chromatogram of a mixed Se standard solution based on HPLC‐ICP‐MS.
**Figure S2** Expression levels of *NRT1.1B* in roots and shoots of the *NRT1.1B*‐overexpressing lines OE‐31/OE‐72 and OEvp‐21/OEvp‐43.
**Figure S3** SeMet content ratios of leaf blades to roots, leaf sheaths to roots and shoots to roots of *NRT1.1B‐*overexpressing lines.
**Figure S4** *NRT1.1B* overexpression up‐regulates *OsPT2* and *OsNIP2.1*.
**Figure S5** Grain yield and 1000‐grain weight assays of *NRT1.1B‐*overexpressing lines OEvp‐21 and OEvp‐43.
