# Supplementary figures and images for: NRT1.1B improves selenium concentrations in rice grains by facilitating selenomethinone translocation
Source: Plant Biotechnol J. 2019 Jan 9;17(6):1058–68. doi: 10.1111/pbi.13037 (PMC6523590; doi:10.1111/pbi.13037)

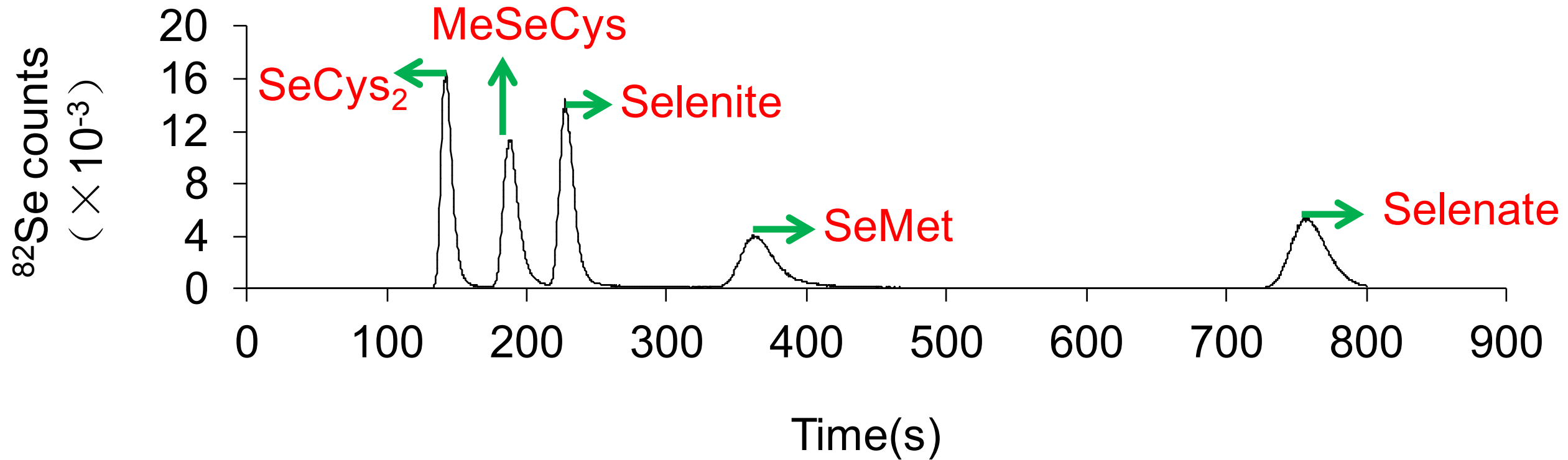

Se standard solution

(a)

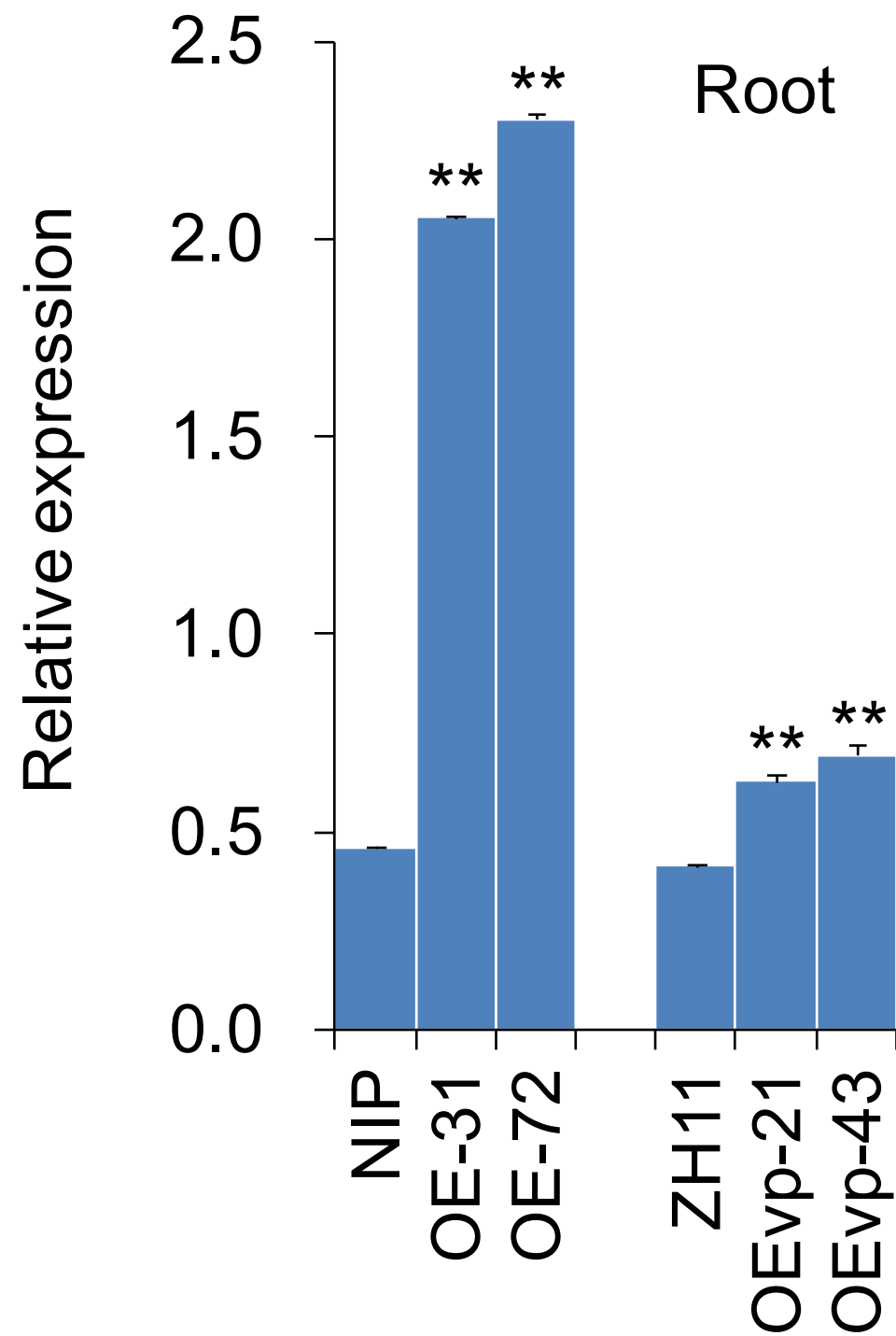

(b)

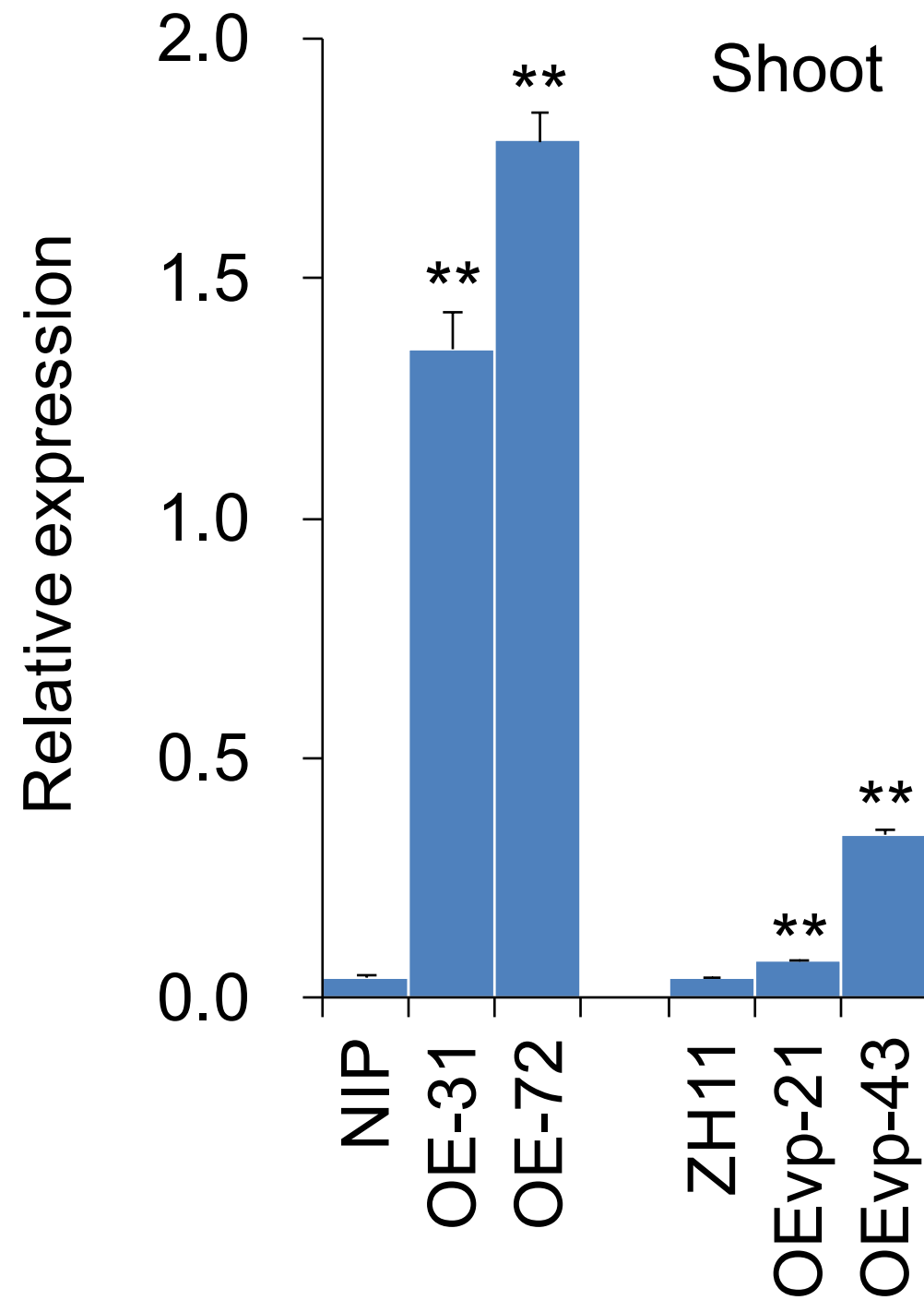

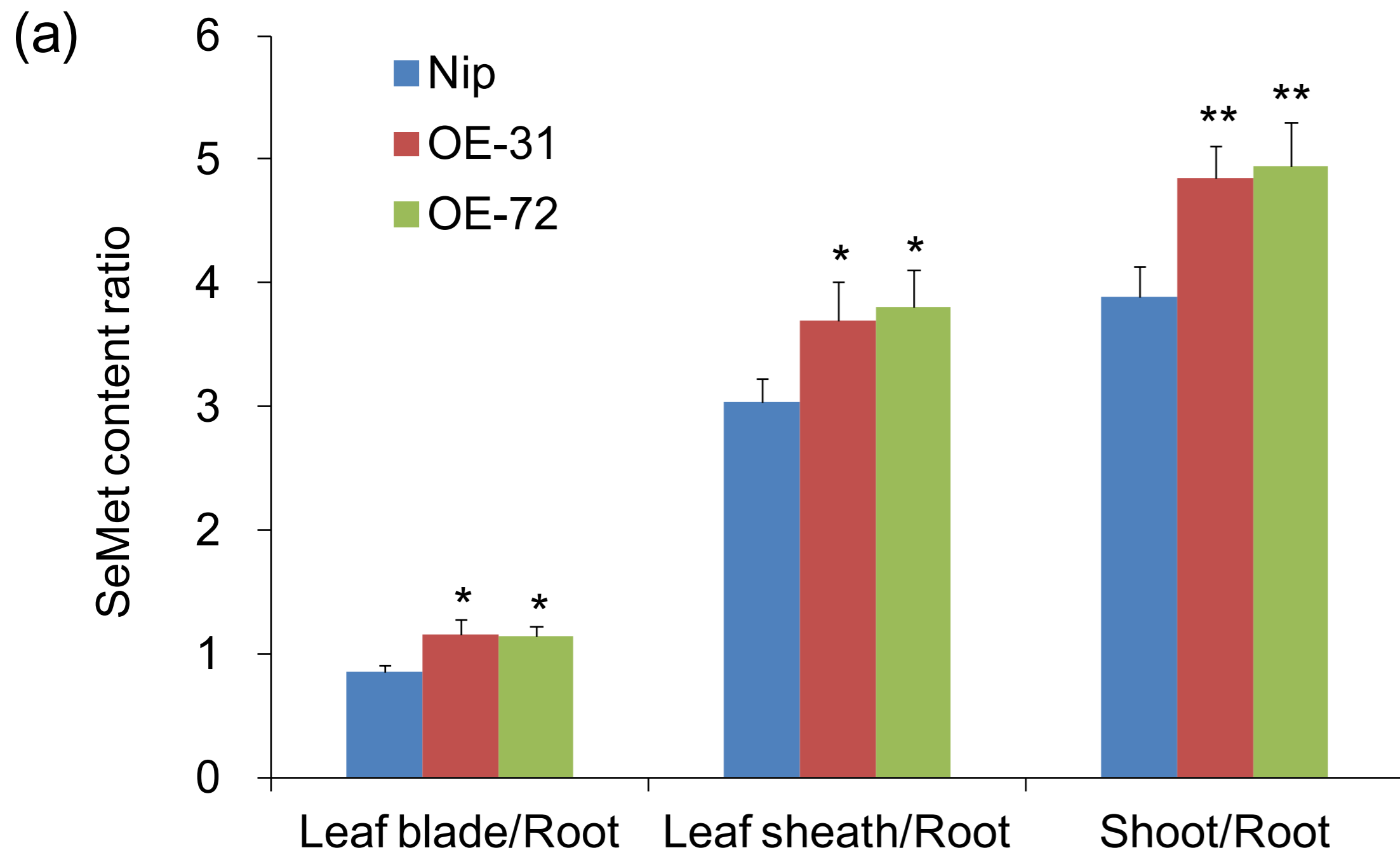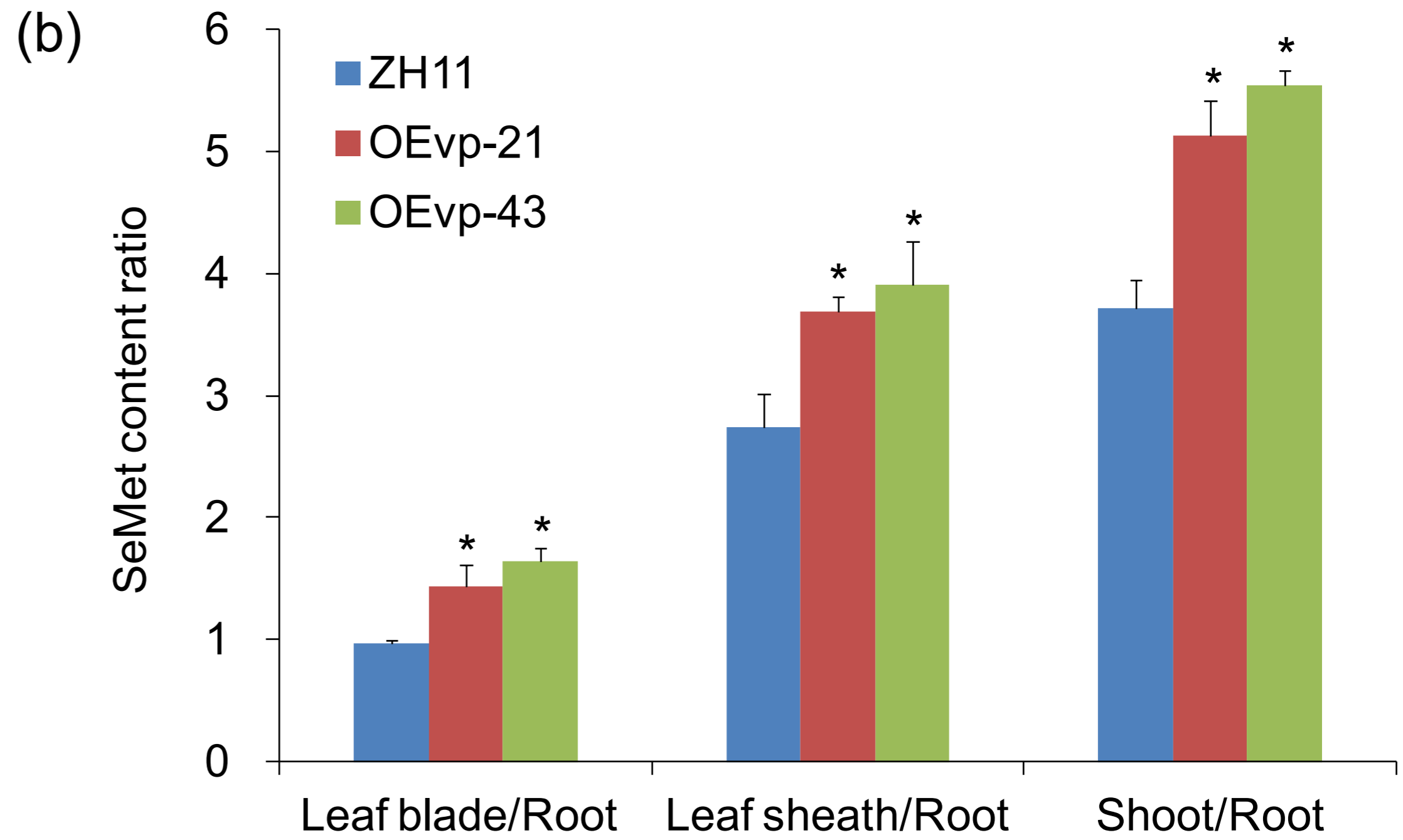

(a)

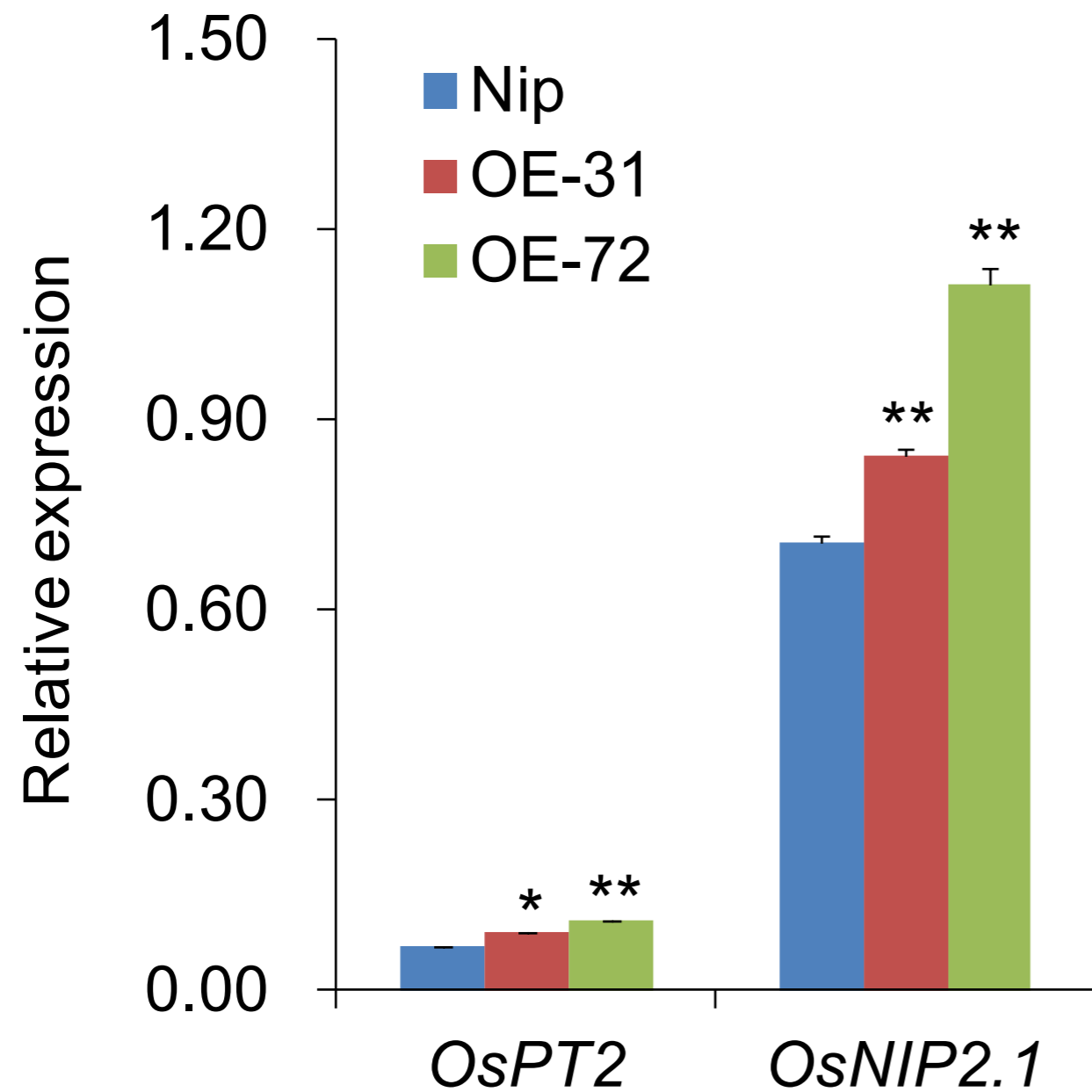

(b)

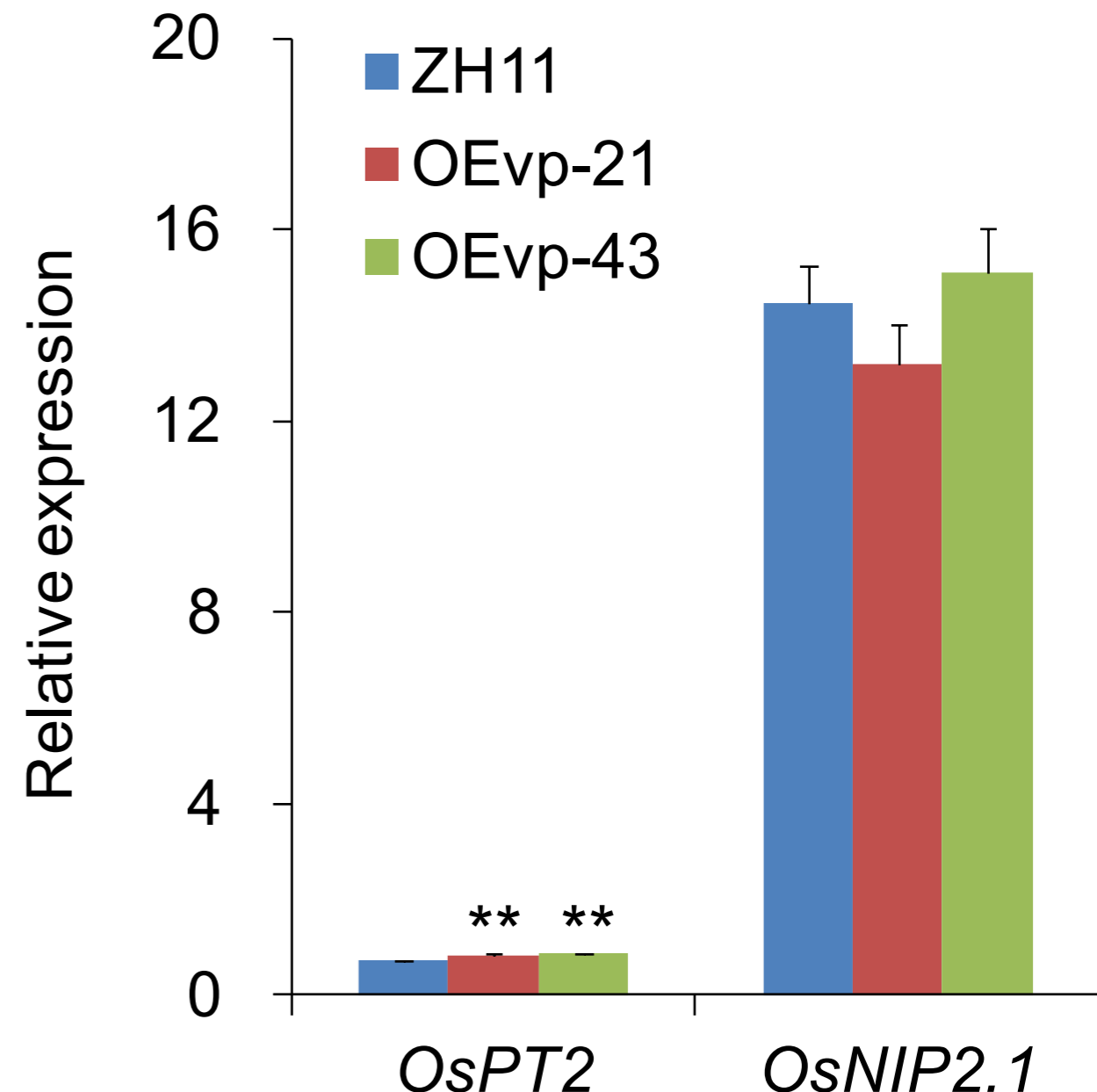

(a)

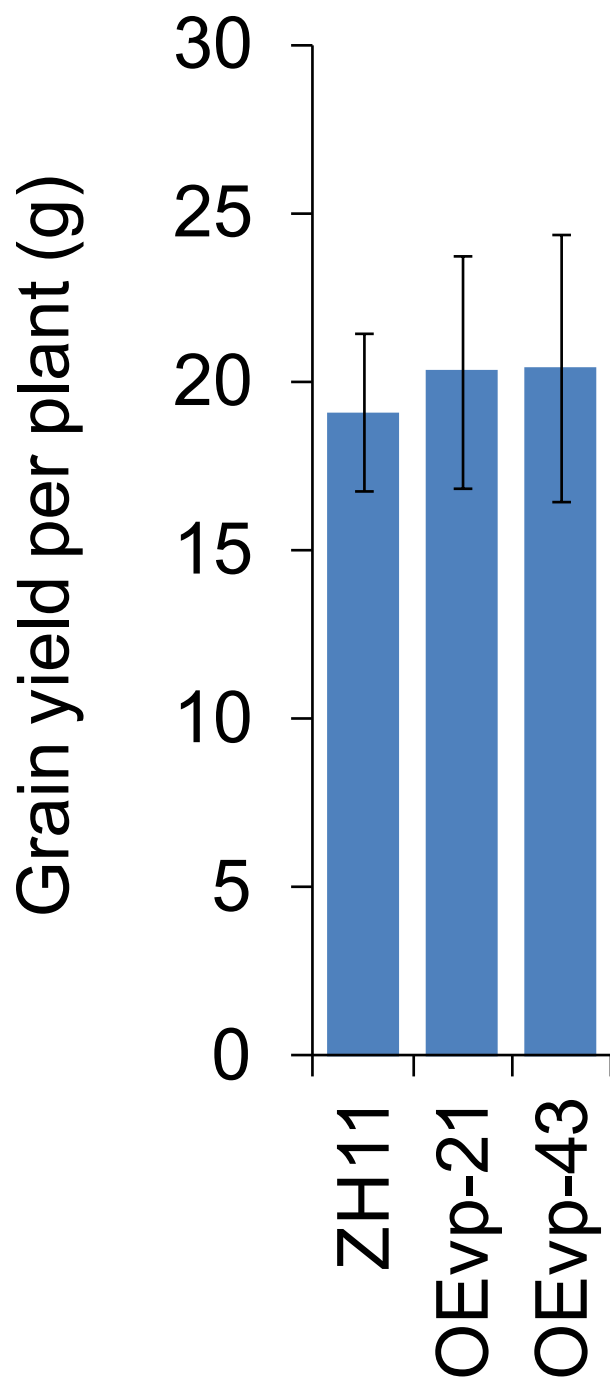

(b)

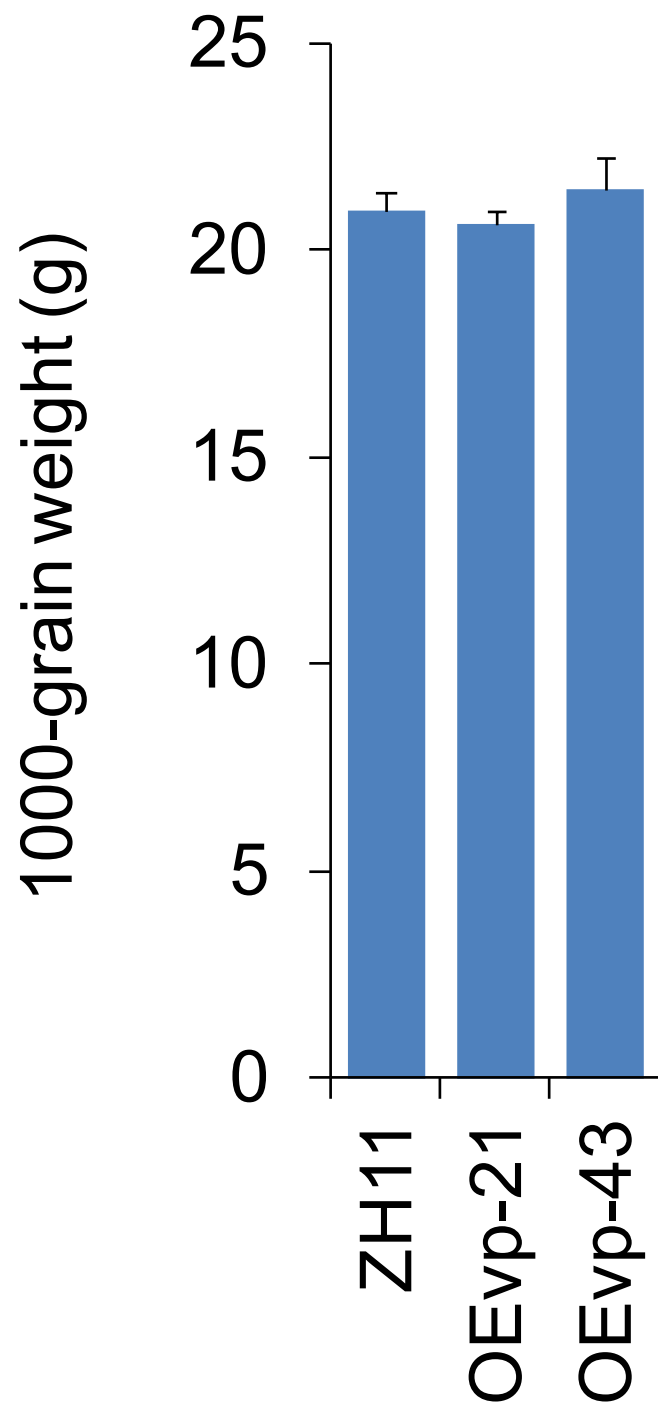

Supplement: Supplementary file 1 — Figure S1 Chromatogram of a mixed Se standard solution based on HPLC‐ICP‐MS. Figure S2 Expression levels of NRT1.1B in roots and shoots of the NRT1.1B‐overexpressing lines OE‐31/OE‐72 and OEvp‐21/OEvp‐43. Figure S3 SeMet content ratios of leaf blades to roots, leaf sheaths to roots and shoots to roots of NRT1.1B‐overexpressing lines. Figure S4 NRT1.1B overexpression up‐regulates OsPT2 and OsNIP2.1. Figure S5 Grain yield and 1000‐grain weight assays of NRT1.1B‐overexpressing lines OEvp‐21 and OEvp‐43. [file PBI-17-1058-s001.pdf]
